# Burden of relapsing-remitting multiple sclerosis on workers in the US: a cross-sectional analysis of survey data

**DOI:** 10.1186/s12883-019-1495-z

**Published:** 2019-10-28

**Authors:** Jacqueline A. Nicholas, Batul Electricwala, Lulu K. Lee, Kristen M. Johnson

**Affiliations:** 1 OhioHealth Multiple Sclerosis Clinic, Riverside Methodist Hospital, Columbus, OH USA; 2 Novartis Pharmaceuticals Corporation, One Health Plaza, East Hanover, NJ 07936 USA; 3 Kantar Health, San Mateo, CA USA

**Keywords:** Absenteeism, Presenteeism, Healthcare resource utilization, Relapsing-remitting multiple sclerosis, Work impairment

## Abstract

**Background:**

Multiple sclerosis (MS) is prevalent among working age individuals (20–60 years), leading to high burden on work productivity. Few data are available about the absenteeism and presenteeism in employed individuals with MS in comparison to non-MS personnel. This study aimed to quantify the burden of illness of employed US adults with relapsing-remitting multiple sclerosis (RRMS) and examine burden by levels of work impairment.

**Methods:**

A retrospective cross-sectional analysis was conducted using patient-reported responses from the US National Health and Wellness Survey (NHWS). Data from NHWS 2015–2016 were analyzed from 196 employed RRMS respondents who were matched 1:4 to employed respondents without MS based on demographic and general health characteristics. Demographic and general health characteristics for employed RRMS individuals were analyzed by levels of work impairment (none, 1–30%; 31–68%; 69–100%). Work productivity (absenteeism, presenteeism, and work impairment), decrements in health-related quality of life (HRQoL) (short form-36, EQ-5D), and healthcare resource utilization (HCRU) were compared to determine the burden of RRMS.

**Results:**

After propensity score matching, the levels of absenteeism and presenteeism were 2 and 1.8 times higher in the employed RRMS population than the employed non-MS population, respectively (*P* < 0.001 for both). HRQoL was significantly lower in employed respondents with RRMS than those without MS (*P* < 0.001 for all). Employed respondents with RRMS had significantly more HCRU over 6 months compared to those without MS (*P* < 0.001). Furthermore, among employed RRMS respondents, greater levels of impairment were associated with increasing disease severity, greater healthcare resource use, fatigue, and cognitive impairment and inversely associated with mental and physical HRQoL (*P* < 0.0001 for all).

**Conclusions:**

Among employed individuals, respondents with RRMS had lower, work productivity, HRQoL, and higher HCRU as compared with those without MS. Given the large impact RRMS has on work impairment, a need exists to manage individuals on therapies that improve HRQoL, reduce symptoms, and improve their ability to perform in the workforce.

## Background

Multiple sclerosis (MS) is a chronic autoimmune, inflammatory, demyelinating disease of the central nervous system [1], characterized by neurological symptoms involving the motor, sensory, visual, and autonomic systems [2]. Symptoms and impairments are variable and include fatigue, difficulty walking, imbalance, numbness, pain, bowel and bladder impairment, sexual dysfunction and cognitive impairment [3]. MS can either be characterized as progressive, secondary progressive or relapsing-remitting (RRMS). RRMS is the most common form [4], and approximately 85% of individuals with MS are initially diagnosed with RRMS. The disease is characterized by flare-ups (relapses or exacerbations) of symptoms followed by period of remission when symptoms improve or disappear [5]. Fortunately, our understanding of the MS has grown in recent years as some genetic and environmental factors have been identified, including low vitamin D levels, cigarette smoking, and obesity [6].

MS is the most common inflammatory neurological disease in young adults [7]. The disease affects at least 2.2–2.3 million people worldwide with prevalence estimates of 50–300 per 100,000 worldwide and approximately 165 per 100,000 population in high-income North America alone (United States [US], Canada, and Greenland) [6–11]. A substantial number of people throughout the world remain undiagnosed, thus the actual prevalence of MS is likely even higher [11]. In many geographic regions the prevalence of MS has increased since 1990 [7]. For example, the prevalence of this disease in the US was estimated at approximately 400,000 individuals in 2016 [10]. These numbers underscore the significant societal burden of the disease.

MS is often diagnosed in early to middle adulthood, most commonly causing disability, fatigue, depression, and anxiety [3, 8], thus affecting the primary productive years of life [12]. Having MS can create barriers to employment and income-earning [1, 12]. MS negatively affects the productivity of individuals during their working careers. One study observed the highest prevalence for MS was in individuals 45 to 49 years of age [10]. However, the average age of disease onset is between the ages of 30 to 35 years [13]. Due to this relatively early age of disease onset, impairment relating to MS can last for decades of a person’s work life. Since most people in the US retire by age 69 [14], this impairment may impact a large proportion of the working life of MS patients. A systematic review of studies conducted in the US and abroad found that early retirement for patients with MS played a large role in the financial burden of their disease [15].

MS can negatively impact an individual’s quality of life (QoL) [16] and work productivity [17, 18] and greatly increase healthcare resource utilization (HCRU) [19]. A large, US cross-sectional general health survey by Gupta et al. found increased MS severity was associated with greater work and activity impairment, diminished health-related quality of life (HRQoL), and increased HCRU [20]. In a cross-sectional survey of physician-recruited RRMS patients, a significant association was found between level of disability and HCRU, but was limited to patients using a disease-modifying therapy continuously for 1 year [21]. Lastly, a prospective observational cohort study, the Comprehensive Longitudinal Investigation of MS at the Brigham and Women’s Hospital, Partner’s MS Center (CLIMB) study, examined work productivity, disability, depression, fatigue, anxiety, cognition, and HRQoL in 377 patients with either clinically isolated syndrome (CIS) or RRMS. The study found substantial decreases in work productivity due to presenteeism (being present, but working in a reduced capacity due to illness or injury) and reduced work productivity. In this study, CIS was not differentiated from RRMS and the study sample was limited in terms of geographic scope. Importantly, this study reported a high rate of employment (76%), which suggests the sample may not be representative of RRMS patients [17]. The results of these studies suggest the need for additional research to specifically characterize work impairment among the RRMS population in the US and reinforce the growing importance and value of assessing presenteeism in any economic evaluation and cost of illness studies [22]. However, there is paucity of data on the detailed impact of RRMS on individuals’ ability to maintain employment.

The objective of the current study was to describe the burden of illness in U.S. adults with RRMS, with a specific focus on those who are currently employed and experiencing work impairment. Relapsing populations make up > 70% of the MS population, hence, this patient population is of great interest to treat for delaying progression. The aim of the study was to examine employment and HRQOL, HCRU in employed individuals with RRMS and without MS and further quantify these outcomes in employed RRMS individuals by level of work impairment.

## Methods

### Data source

A retrospective analysis was conducted using an existing database of responses to the National Health and Wellness Survey (NHWS), a self-reported cross-sectional survey designed to reflect the general US population including individuals who report diagnosis of MS. The NHWS is an internet-based general health questionnaire distributed to a sample of the adult population. Respondents were qualified if they were ≥ 18 years of age, able to read and write English, and electronically provided informed consent. Respondents were recruited through opt-in email, co-registration with MySurvey.com partners, eNewsletter campaigns, banner placements, and both internal and external affiliate networks, using a stratified random sampling framework to ensure representativeness of the US population in terms of age and gender. Additional details about the NHWS have previously been published [23–25].

### Sample selection

Data from 2015 to 2016 (2015 NHWS, *N* = 97,700 and 2016 NHWS, *N* = 97,503; total *N* = 195,203) were analyzed. If an individual completed the survey in both years, the response in the most recent year was used. Respondents who reported being diagnosed with MS by a doctor and indicated RRMS as the type of MS were included in the RRMS group. Potential control respondents were selected from those who self-reported as not having a diagnosis of MS.

### Measures

The following patient and disease characteristics were evaluated: age, gender, employment status, annual household income, marital status, education, possession of health insurance, body mass index (BMI), smoking status, alcohol use, exercise, Charlson comorbidity index score (CCI) [26], and emotional issues such as anxiety and depression, and sleep problems. The CCI allows for adjustment of baseline comorbidity between groups, and is a widely used comorbidity index in studies that use administrative health data [27]. The higher the score, the more likely the predicted outcome will result in mortality or higher resource use [28, 29]. MS characteristics included severity of MS, symptoms, fatigue, and perceived cognitive impairment.

The HRQOL was measured using Short Form (SF)-36v2 and EQ-5D. In SF-36v2, the HRQoL was captured by the physical (PCS) and mental (MCS) component summary scores [30, 31] Both the MCS and PCS have a theoretical range of 0–100 [25]. Higher scores on these measures indicate better HRQoL. The EQ-5D was used as a utility measure of health and was expressed as a health utility index score [25]. Previously, minimally important differences (MIDs) have been defined by differences of 5.0 points for MCS and PCS scores and 0.074 for the EQ-5D [25, 31–33]. The impact on labor force participation was measured by defining employment status as currently in the labor force (full-time employed, part-time employed, self-employed, or not unemployed but looking for work) or not currently in the labor force (retired, disabled, not employed and not looking for work). The Work Productivity and Activity Impairment-General Health scale (WPAI-GH) assessed work productivity loss and activity impairment [34]. The WPAI-GH contains six questions [35, 36]. The WPAI-GH captures absenteeism (% of work time missed because of one’s health), presenteeism (% impairment while at work because of one’s health), overall work impairment (% of overall work impairment due to health; a combination of absenteeism and presenteeism), and activity impairment (% of impairment in daily activities because of one’s health) [35]. WPAI outcomes are expressed as impairment percentages, with higher numbers indicating greater impairment and less productivity. Absenteeism, presenteeism, and overall work impairment were calculated for employed respondents only, whereas activity impairment was calculated for all respondents.

HCRU included visits to healthcare providers (HCPs), general physician (GP) or primary care physician (PCP), specialists (e.g., neurologists), emergency rooms (ER) and hospitalizations in the prior 6 months.

### Statistical analysis

#### Bivariate Ana006Cyses

Two-sample comparisons using Chi-square tests for categorical variables and one-way ANOVAs for continuous and count variables were conducted between employed respondents diagnosed with RRMS and those not diagnosed with MS to characterize the two populations and determine baseline variables for propensity score matching.

#### Propensity score matching

Propensity score matching is used to obtain similar groups of treatment and control subjects by matching individual observations on their propensity scores [37]. Employed individuals who reported a diagnosis of RRMS were propensity matched to employed individuals without a diagnosis of MS at a 1:4 ratio based on the survey year, age, gender, education, type of health insurance, BMI, and comorbidity burden as assessed by the CCI. These demographic and patient characteristics were included as criteria for the propensity score match in order to control for differences between the two groups. Balance post propensity match was examined using ANOVA, chi-square tests and *p*-values for variables which were significant at > 0.05 were considered to not be balanced.

Variables included in the match were entered into a logistic regression to predict presence of RRMS (vs. no MS) and propensity scores were saved from this model to match each individual with RRMS to four individuals without MS using a greedy-matching algorithm. This identified controls to match to a single case at up to eight decimal places of the propensity score (and as little as one decimal place, if no other suitable control was identified) [38, 39].

#### Matched analyses

Bivariate analyses using Chi-square tests for categorical variables and one-way ANOVAs for continuous variables were conducted for the employed RRMS vs. no MS groups on patient characteristics to determine whether balance was achieved post-match. Then, outcomes (e.g., SF-36, EQ5D, WPAI, HCRU) were compared between groups (employed RRMS vs. no MS) using one-way ANOVAs. This was repeated in an analysis comparing RRMS and non-MS controls.

An additional analysis was conducted among employed RRMS individuals. MS characteristics, symptoms, and outcomes (e.g., SF-36, EQ5D, WPAI, HCRU) were described by level of work impairment. Levels of work impairment were defined by tertiles based on the observed distribution of the response variable. Chi-square tests (for categorical variables) and one-way ANOVAs (for continuous variables) were used to compare demographics, health characteristics, and health and economic outcomes by levels of work impairment. All multiple pairwise comparisons were conducted with *t*-tests (continuous variables) or z-tests of column proportions (categorical variables) and adjusted using the Bonferroni correction. *P* < 0.05 between groups is considered as the level of significance. All analyses were performed using SPSS 23.0 and SAS 9.4.

## Results

A total of 176,768 unique respondents completed the US NHWS from 2015 to 2016; of which, 543 indicated a diagnosis of RRMS and 196 were employed. Additional file 1: Figure S1 depicts the selection process for this study.

### RRMS vs non-MS respondents

After matching for demographic and health characteristics, the mean age was 45.2 years for employed RRMS respondents and 45.3 years for non-MS respondents (*P* = 0.971; Table 1). Female preponderance was 69.9% for employed RRMS respondents and 70.5% for non-MS respondents (*P* = 0.861;). High proportions of respondents had health insurance (94.4% of employed RRMS and 95.2% of employed without MS).

**Table 1:** Demographics and general health characteristics of employed RRMS vs. non-MS respondents

	Employed: RRMS Vs. non-MS respondents		Employed: RRMS Vs. non-MS respondents (Matched sample: ratio 1:4)			
	Employed non-MS respondents (*N* = 102,139)	Employed RRMS respondents (*N* = 196)	Employed non-MS respondents (*N* = 784)	Employed RRMS respondents (*N* = 196)	Test result significance^a^	Standardized difference
Age, years (mean [SD])	41.7 (13.9)	45.2 (11.0)	45.3 (11.2)	45.2 (11.0)	0.971	0.009
Gender						
Female, n (%)	51,141 (50.1)	137 (69.9)	553 (70.5)	137 (69.9)	0.861	0.003
Household Income per year, n (%)						
< $25 k	11,873 (11.6)	23 (11.7)	88 (11.2)	23 (11.7)	0.487	
$25–< 50 k	23,517 (23.0)	41 (20.9)	161 (20.5)	41(20.9)		
50–< 75 k	21,029 (20.6)	44 (22.4)	169 (21.6)	44 (22.4)		
> $75 k	41,480 (40.6)	84 (42.9)	326 (41.6)	84 (42.9)		
Declined to answer	4240 (4.2)	4 (2.0)	40 (5.1)	4 (2.0)		
Marital Status, n (%)						
Married or living with partner	58,213 (57.0)	125 (63.8)	491 (62.6)	125 (63.8)	0.766	
Not	43,650 (42.7)	71 (36.2)	293 (37.4)	71 (36.2)		
Declined to answer	276 (0.3)	0 (0)	–	–		
Level of Education, n (%)						
Completed university education	55,295 (54.1)	100 (51.0)	394 (50.3)	100 (51.0)	0.769	0.0041
Not	46,703 (45.7)	96 (49.0)	388 (49.5)	96 (49.0)		0.0044
Declined to answer	141 (0.1)	0 (0)	2 (0.3)	0 (0)		N/A
Health Insurance, n (%)						
Yes	91,509 (89.6)	185 (94.4)	746 (95.2)	185 (94.4)	0.660	
No	10,630 (10.4)	11 (5.6)	38 (4.8)	11 (5.6)		0.0378
Body Mass Index, n (%)						
Underweight (< 18.5 kg/m2)	3244 (3.2)	6 (3.1)	23 (2.9)	6 (3.1)	0.122	0.076
Normal weight (18.5 to < 25.0 kg/m2)	35,823 (35.1)	74 (37.8)	232 (29.6)	74 (37.8)		0.0051
Overweight (25 to < 30.0 kg/m2)	31,519 (30.9)	48 (24.5)	251 (32.0)	48 (24.5)		0.0094
Obese (30.0 kg/m2 and above)	28,223 (27.6)	63 (32.1)	246 (31.4)	63 (32.1)		0.0066
Declined to answer	3330 (3.3)	5 (2.6)	32 (4.1)	5 (2.6)		0.1055
Smoking Status, n (%)						
Current	16,893 (16.5)	49 (25.0)	196 (25.0)	49 (25.0)	1.000	
Former	21,895 (21.4)	52 (26.5)	208 (26.5)	52 (26.5)		
Never	63,351 (62.0)	95 (48.5)	380 (48.5)	95 (48.5)		
Use of Alcohol, n (%)						
Yes	74,820 (73.3)	138 (70.4)	202 (25.8)	58 (29.6)	0.278	
No	27,319 (26.7)	58 (29.6)	582 (74.2)	138 (70.4)		
Vigorous Exercise in the Past 30 Days, n (%)						
Yes	73,040 (71.5)	132 (67.3)	263 (33.5)	64 (32.7)	0.813	
No	29,099 (28.5)	64 (32.7)	521 (66.5)	132 (67.3)		
Comorbid Medical Conditions, n (%)						
Experienced Anxiety in the past 12 months	34,639 (33.9)	88 (44.9)	271 (34.6)	88 (44.9)	0.007	
Experienced Depression in the past 12 months	24,914 (24.4)	71 (32.6)	204 (26.0)	71 (36.2)	0.004	
Experienced sleep problems in the past 12 months	31,473 (30.8)	87 (44.4)	263 (33.5)	87 (44.4)	0.004	
CCI Scoring, mean (SD)	0.29 (0.83)	0.44 (1.40)	0.34 (0.82)	0.44 (1.40)	0.174	0.103

*CCI* Charlson Comorbidity Index, *MS* Multiple Sclerosis, *RRMS* Relapsing Remitting Multiple Sclerosis, *SD* Standard Deviation

^a^Significance from Pearson chi-square tests except for age, which was from one-way ANOVA

### Employed RRMS vs non-MS respondents

The analysis of the employed respondents (RRMS vs non-MS controls) showed significant differences in terms of health status, work impairment, and HCRU (Table 2). Comparison of outcomes revealed significantly lower health status among respondents with RRMS relative to the matched controls not diagnosed with MS, with a difference of 2.4 points on MCS, 7.8 points on PCS, and 0.1 points on the EQ-5D index (*P* ≤ 0.001 for all) between the two groups. The analysis indicated that employed respondents with RRMS had substantial work productivity impairment (37.0 vs. 20.4, *P* < 0.001; Table 2), and total activity impairment (38.7 vs. 20.3, *P* < 0.001) compared with matched employed controls without MS. The levels of absenteeism and presenteeism among employed respondents with RRMS were approximately 2- (12.3 vs. 6.3, *P* < 0.001) and 1.8-fold (33.4 vs. 18.6, *P* < 0.001) higher than the matched respondents without MS, respectively (*P* < 0.001). In terms of HCRU, respondents with RRMS reported significantly higher HCP and neurologist visits over last 6 months of the completed survey relative to matched controls (*P* < 0.001 for both). The number of GP/PCP visits were not significantly different between the two groups.

**Table 2:** Health status, work impairment, healthcare use in employed RRMS vs. non-MS respondents

Parameters	Employed RRMS respondents (*N* = 196)	Employed: non-MS respondents (*N* = 784)	*P* value
MCS, mean ± SD	44.80 ± 10.74	47.24 + 10.36	0.004
PCS, mean ± SD	44.04 ± 9.04	51.80 ± 8.44	< 0.001
EQ5D Index, mean ± SD	0.72 ± 0.18	0.85 ± 0.14	< 0.001
Absenteeism, mean ± SD	12.27 ± 24.36	6.28 ± 17.22	< 0.001
Presenteeism, mean ± SD	33.43 ± 29.63	18.61 ± 27.28	< 0.001
Total work productivity impairment, mean ± SD	36.95 ± 32.87	20.41 ± 29.76	< 0.001
Total activity impairment, mean ± SD	38.72 ± 30.58	20.26 ± 27.90	< 0.001
Total number of HCP visits in the past 6 months, mean ± SD	5.48 ± 9.33	3.27 ± 4.80	< 0.001
GP/PCP number of visits in the past 6 months, mean ± SD	1.07 ± 1.61	0.94 ± 1.40	0.269
Neurologist number of visits in the past 6 months, mean ± SD	0.93 ± 1.03	0.05 ± 0.37	< 0.001

*HCP* Health Care Provider, *GP* General Physician, *PCP* Primary Care Physician, *PCS* Physical Component Summary, *MCS* Mental Component Summary, *MS* Multiple Sclerosis, *RRMS* Relapsing Remitting Multiple Sclerosis, *SD* Standard Deviation, *vs* versus

### Employed respondents with RRMS by levels of work impairment

Analyses among employed respondents with RRMS by levels of work impairment showed those with greater work impairment were less likely to have exercised vigorously within 30 days of completing the survey (*P* = 0.001; Table 3). Those with greater work impairment reported significantly more CCI comorbidities (*P* = 0.03) than those with less work impairment. Other demographic and general health characteristics (age, gender, employment status, household income, marital status, level of education, possession of health insurance, BMI, smoking status, use of alcohol) were not significantly affected by levels of work impairment.

**Table 3:** Demographics and general health characteristics of employed RRMS respondents by levels of work impairment

Parameters	No work impairment (*N* = 47)	1–30% (*N* = 48)	31–68% (*N* = 42)	69–100% (*N* = 44)	*P* value
Age, years (mean ± SD)	47.6 ± 10.6	46.5 ± 12.8	44.2 ± 9.5	41.95 ± 11.3	0.08
Gender, Female (%)	83	68.8	69	56.8	0.06
Employment status, % (n)					
Yes	100 (47)	100 (48)	100 (42)	100 (44)	
Household income per year, % (n)					
< $25 k	8.5 (4)	10.4 (5)	11.9 (5)	11.4 (5)	0.289
$25- < 50 k	21.3 (10)	14.6 (7)	28.6 (12)	22.7 (10)	
50- < 75 k	29.8 (14)	18.8 (9)	21.4 (9)	20.5 (9)	
≥ $75 k	38.3 (18)	56.3 (27)	31.0 (13)	45.5 (20)	
Declined to answer	2.1 (1)	0.0 (0)	7.1 (3)	0.0 (0)	
Marital status, % (n)					
Married/Living with partner	59.6 (28)	68.8 (33)	61.9 (26)	68.2 (30)	0.738
Not married	40.4 (19)	31.3 (15)	38.1 (16)	31.8 (14)	
Level of education, % (n)					
Completed University Education	46.8 (22)	58.3 (28)	45.2 (19)	52.3 (23)	0.583
Possession of health insurance, % (n)					
Yes	93.6 (44)	97.9 (47)	97.6 (41)	88.6 (39)	0.185
Body mass index, kg/m^2,^ % (n)					
< 18.5	2.1 (1)	0.0 (0)	7.1 (3)	4.5 (2)	0.576
18.5 - < 25	42.6 (20)	41.7 (20)	23.8 (10)	43.2 (19)	
25 - < 30	21.3 (10)	22.9 (11)	35.7 (15)	20.5 (9)	
≥ 30	31.9 (15)	33.3 (16)	28.6 (12)	29.5 (13)	
Declined to Answer	2.1 (1)	2.1 (1)	4.8 (2)	2.3 (1)	
Smoking status, % (n)					
Current	19.1 (9)	18.8 (9)	26.2 (11)	38.6 (17)	0.220
Former	29.8 (14)	29.2 (14)	33.3 (14)	15.9 (7)	
Never	51.1 (24)	52.1 (25)	40.5 (17)	45.5 (20)	
Use of alcohol, % (n)					
Yes	70.2 (33)	66.7 (32)	59.5 (25)	84.1 (37)	0.085
Vigorous exercise in the past 1 month, % (n)					
Yes	87.2 (41)	58.3 (28)	52.4 (22)	75.0 (33)	< 0.001
Comorbid medical conditions, % (n)					
Experienced anxiety (last 1 year)	25.5 (12)	33.3 (16)	52.4 (22)	70.5 (31)	< 0.001
Experienced depression (last 1 year)	19.1 (9)	27.1 (13)	42.9 (18)	59.1 (26)	< 0.001
Experienced sleep problems (last 1 year)	27.7 (13)	43.8 (21)	57.1 (24)	50.0 (22)	0.034
CCI score, mean ± SD	0.19 ± 0.54	0.35 ± 1.12	0.36 ± 0.62	1.05 ± 2.51	0.025

*CCI* Charlson Comorbidity Index, *n* count, *RRMS* Relapsing Remitting Multiple Sclerosis

**Fig. 1:**
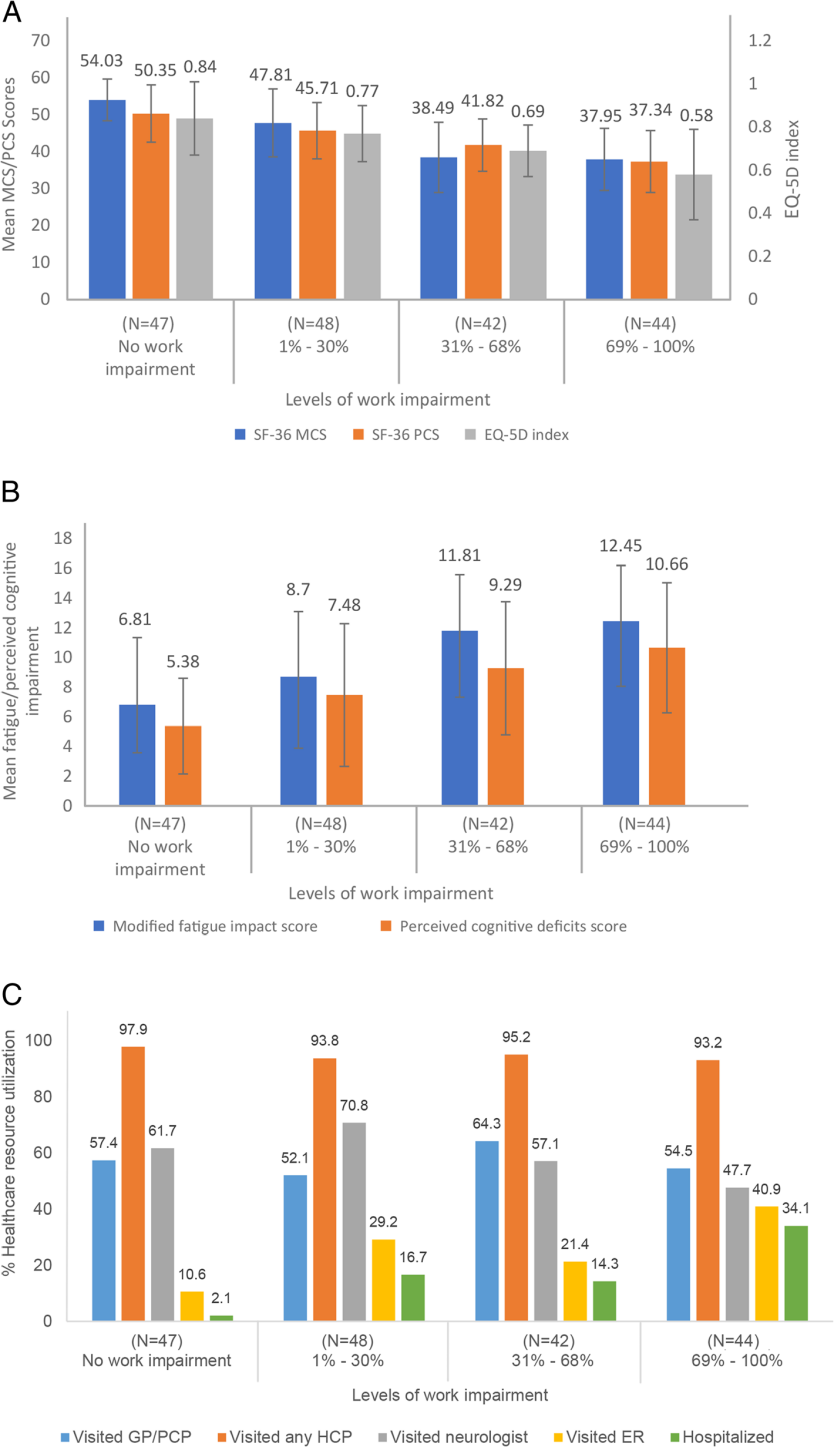
**a** Health-Related Quality of Life Among Employed RRMS Respondents. All values are expressed as mean ± SD. *Overall omnibus *P* value is < 0.001. EQ-5D, EuroQoL-5 dimension; MCS, Mental Component Summary; PCS, Physical Component Summary; RRMS, Relapsing-Remitting Multiple Sclerosis. **b** Fatigue/Perceived Cognitive Impairment Among Employed RRMS Respondents. All values are expressed as mean ± SD. *Overall omnibus P value is < 0.001. RRMS, Relapsing-Remitting Multiple Sclerosis; SD, Standard Deviation. **c** Percent Healthcare Resource Utilization Among Employed RRMS Respondents. All values are expressed as % *P* < 0.05 for neurologist visits, ER visits, and hospitalizations. GP, General Physician; HCP, Healthcare Provider; PCP, Primary Care Physician; RRMS, Relapsing-Remitting Multiple Sclerosis; SD, Standard Deviation

HRQoL (SF-36 MCS, SF-36 PCS, EQ-5D indexes), fatigue, perceived cognitive impairment, and HCRU by level of work impairment are shown in Fig. 1a. Employed respondents with RRMS with greater work impairment reported significantly worse scores on QoL measures, including the MCS, PCS, and EQ-5D (Fig. 1a, *P* < 0.001 for all). Further, these respondents reported significantly greater fatigue and perceived cognitive deficits (Fig. 1b *P* < 0.001 for both), and reported greater HCRU, specifically more ER visits, and hospitalizations over the last 6 months before completing the survey, compared to those with less work impairment (*P* < 0.001 for all; Fig. 1c).

The analyses of the employed RRMS respondents by levels of work impairment showed significant effects in terms of health status, work impairment, fatigue/perceived cognitive impairment, and HCRU (Table 4). Significant decreases in MCS, PCS, and EQ5D index scores were found compared with those RRMS respondents with no work impairment (all *P* < 0.001). In general, the greater the level of work impairment, the lower the score for these three parameters. Absenteeism, presenteeism, total work productivity impairment, total activity impairment, modified fatigue impact score, perceived cognitive deficits score, HCP visits, and GP/PCP visits were found to increase for RRMS respondents with the level of work impairment compared with those who reported no work impairment (*P* ≤ 0.04). No significant effect was observed on the number of neurologist visits by the level of work impairment.

**Table 4:** Work impairment, fatigue/perceived cognitive impairment, and healthcare usage among employed RRMS respondents

Parameters	No work impairment (*N* = 47)	1–30% (*N* = 48)	31–68% (*N* = 42)	69–100% (*N* = 44)	*P* value
MSS, mean ± SD	54.03 ± 5.63	47.81 ± 9.22	38.49 ± 9.54	37.95 ± 8.43	< 0.001
PSC, mean ± SD	50.35 ± 7.76	45.71 ± 7.63	41.82 ± 7.07	37.34 ± 8.39	< 0.001
EQ5D index. Mean ± SD	0.84 ± 0.17	0.77 ± 0.13	0.69 ± 0.12	0.58 ± 0.21	< 0.001
Absenteeism, mean ± SD	0.00 ± 0.00	0.65 ± 3.69	5.60 ± 10.41	36.45 ± 27.35	< 0.001
Presenteeism, mean ± SD	0.00 ± 0.00	18.13 ± 8.16	46.67 ± 9.02	73.18 ± 15.96	< 0.001
Total work productivity impairment, mean ± SD	0.00 ± 0.00	18.75 ± 7.82	49.83 ± 8.86	83.98 ± 9.67	< 0.001
Total activity impairment, mean ± SD	5.11 ± 12.14	24.38 ± 18.55	54.76 ± 13.48	75.68 ± 13.54	< 0.001
Modified fatigue impact score, mean ± SD	6.81 ± 4.53	8.7 ± 4.41	11.81 ± 3.78	12.45 ± 3.75	< 0.001
Perceived cognitive deficits score, mean ± SD	5.38 ± 3.22	7.48 ± 4.8	9.29 ± 4.48	10.66 ± 4.39	< 0.001
Total number of HCP visits in the past 6 months, mean ± SD	3.21 ± 2.37	3.94 ± 2.76	6.60 ± 15.69	8.41 ± 10.59	0.03
GP/PCP number of visits in the past 6 months, mean ± SD	0.77 ± 1.03	0.69 ± 0.78	1.29 ± 1.55	1.39 ± 2.09	0.04
Neurologist number of visits in the past 6 months, mean ± SD	0.77 ± 0.73	1.00 ± 0.90	0.98 ± 1.16	0.84 ± 1.10	0.62

*HCP* Health Care Provider, *GP* General Physician, *MSS* Mental Summary Scores, *PCP* Primary Care Physician, *PSC* Physical Summary Scores, *RRMS* Relapsing Remitting Multiple Sclerosis, *SD* Standard Deviation

Among employed respondents with RRMS, MS symptom severity was directly related to the degree of work impairment. Overall, the respondents with greater work impairment reported a significantly higher MS severity than respondents with less work impairment (*P* < 0.001). Among respondents with substantial levels of work impairment (> 30%), the most commonly reported MS symptoms included fatigue, pain, numbness, difficulty with balance, and difficulty with concentrating and remembering (Table 5).

**Table 5:** MS symptoms of employed RRMS respondents by levels of work impairment

MS symptoms	Currently have following MS symptoms			
	No work impairment (*N* = 47)	1–30% (*N* = 48)	31–68% (*N* = 42)	69–100% (*N* = 44)
Breathing problems, % (n)	2.3 (1)	0.0 (0)	9.8 (4)	7.1 (3)
Constipation, % (n)	18.2 (8)	10.6 (5)	12.2 (5)	26.2 (11)
Difficulty in concentrating, % (n)	20.5 (9)	25.5 (12)	51.2 (21)	33.3 (14)
Diarrhea, % (n)	4.5 (2)	2.1 (1)	19.5 (8)	9.5 (4)
Depression, % (n)	13.6 (6)	12.8 (6)	29.3 (12)	31 (13)
Difficulty remembering, % (n)	31.8 (14)	25.5 (12)	61 (25)	40.5 (17)
Difficulty with speech, % (n)	4.5 (2)	4.3 (2)	24.4 (10)	11.9 (5)
Difficulty balancing or walking, % (n)	36.4 (16)	44.7 (21)	41.5 (17)	45.2 (19)
Dizziness, % (n)	11.4 (5)	6.4 (3)	34.1 (14)	23.8 (10)
Fatigue, % (n)	54.5 (24)	55.3 (26)	75.6 (31)	59.5 (25)
Hearing loss, % (n)	6.8 (3)	0 (0)	7.3 (3)	9.5 (4)
Irritability, % (n)	13.6 (6)	8.5 (4)	34.1 (14)	31 (13)
Itching, % (n)	4.5 (2)	8.5 (4)	12.2 (5)	14.3 (6)
Muscle spasms, % (n)	27.3 (12)	25.5 (12)	48.8 (20)	38.1 (16)
Mood swings, % (n)	11.4 (5)	4.3 (2)	24.4 (10)	31.0 (13)
Numbness of face, body, arms or legs, % (n)	45.5 (20)	34 (16)	58.5 (24)	38.1 (16)
None of the above, % (n)	18.2 (8)	10.6 (5)	4.9 (2)	4.8 (2)
Pain, % (n)	27.3 (12)	29.8 (14)	56.1 (23)	45.2 (19)
Sexual dysfunction, % (n)	13.6 (6)	10.6 (5)	19.5 (8)	28.6 (12)
Swallowing problems, % (n)	11.4 (5)	6.4 (3)	9.8 (4)	16.7 (7)
Stiffness, % (n)	15.9 (7)	19.1 (9)	48.8 (20)	31 (13)
Seizures, % (n)	0	2.1 (1)	0	4.8 (2)
Tremor, % (n)	9.1 (4)	4.3 (2)	17.1 (7)	9.5 (4)
Urinary incontinence or urgency, % (n)	22.7 (10)	21.3 (10)	26.8 (11)	33.3 (14)
Vision problems, % (n)	22.7 (10)	14.9 (7)	31.7 (13)	19 (8)

*MS* Multiple Sclerosis, *n* count, *RRMS* Relapsing Remitting Multiple Sclerosis

## Discussion

The NHWS was used to examine patient-reported health outcomes, work impairment, and HCRU among employed respondents with RRMS and those without MS in the US. The current study showed that employed individuals with RRMS exhibited greater work impairment, HCRU, and lower HRQoL compared to those without MS. In a previous study, the level of work impairment due to MS was similar to the findings in this study [20]. However, to the best of our knowledge, this is the first study to evaluate outcomes (HRQOL, HCRU) at different levels of work impairment (i.e., based on a tertile distribution: 0%, 1–30%, 31–68%, 69–100%) in individuals with RRMS who are in the workforce.

An earlier study of patients with MS identified from 1998 to 2009 demonstrated the negative impact of MS on HRQoL and reported that on average an MS patient lost 10.04 quality-adjusted life years due to their disease [40]. The present study also highlighted the impact of RRMS on HRQoL. In fact, the minimal important differences (MIDs) of 0.07 points on the EQ-5D index and 3 points on the SF-36 PCS were exceeded in the current study, indicating the magnitude of these influential effects [31, 41].

We found that only 36.1% of the surveyed respondents with RRMS were employed at the time of the study with an average age of 45.2 years. A progressive disease course and increasing age have previously been shown to be associated with unemployment in MS. [12] Large-scale evaluations of the real-world association between RRMS and employment and productivity in the workplace are lacking. Though previous studies have demonstrated the association of MS with considerable rates of unemployment [42, 43], studies have found that absenteeism and presenteeism were both common among individuals with MS. [17] The affected pattern of total work productivity impairment was consistent with absenteeism and presenteeism, with significantly higher levels of impairment among those with RRMS compared with matched controls in the current study. These findings replicate earlier findings about the affected pattern of overall work impairment being consistent with absenteeism and presenteeism [17, 20]. A study by Kigozi et al. found that the influence of disease on presenteeism in employed individuals is high and should considered in economic studies [22]. The impact of RRMS on work productivity appears to be similar to that of patients suffering from other chronic diseases using the NHWS. Absenteeism and presenteeism was reported to be 4.3 and 32.4% in irritable bowel syndrome [44], 5.0 and 20.0% in asthma [45], and 18.3 and 40.5% in Crohn’s disease [46], respectively.

Fatigue, cognitive dysfunction, depression, and impaired mobility have previously been reported to be associated with QoL and thereby unemployment in patients with MS. [47] Our study in respondents with RRMS reiterates that MS symptom severity parallels greater work impairment. These individuals also experience significant decreases in HRQoL indicators including pain, depression, fatigue, and other cognitive impairments. A few longitudinal studies of patients with MS regressing from employed to unemployed status have shown that decreases in cognition and motor functioning are the critical factors [48, 49]. The results of the current study are validated by the real-world data used and add to our understanding regarding the management of RRMS on long-term productivity loss.

Reduced PCS, MCS, and EQ5D scores indicated that for employed respondents with RRMS, both physical and emotional problems (e.g., anxiety and depression) are associated with reduced work productivity. In this study, greater work impairment among respondents with RRMS was associated with significantly more HCP visits, PCP visits, ER visits, and hospitalizations during the previous 6 months compared to the visits and hospitalizations required from those with less work impairment. Appropriate treatment with an efficacious agent should improve MS symptoms, reduce absenteeism/presenteeism, and therefore increase work productivity for individuals with RRMS.

Healthcare costs in MS are driven by the use of disease modifying treatments (DMTs), which are prescribed based on the initial severity of MS and on its subsequent progression [50]. Moccia et al. [50] found that patients who received more expensive DMTs, specifically indicated for a more aggressive disease progression, presented better long-term outcomes (such as lower risk of reaching milestones of short- and long-term disease progression) compared with patients with relatively milder symptoms who received lower-cost DMTs. This issue should be considered not only by physicians when assessing patients with MS to design the most suitable course of treatment, but also by policy makers when establishing eligibility criteria for DMTs [50, 51]. These more expensive DMTs could have beneficial effects on the MS patients’ ability to work, which could be evaluated in further studies.

There are limitations in the current study and they are as follows. The cross-sectional study design allows the detection of association between the variables at a single point in time, but limits causal inferences. Study data were obtained through online self-report, increasing the chances of confounding self-reporting bias. For example, cognitive impairments were perceived by the respondents and not quantified by objective measures of cognition. It was not possible to confirm the patient-reported responses. To overcome this shortcoming, future research could supplement self-reporting with more objective sources of data (e.g., medical records) to validate participants’ responses. Recall bias may have been introduced, due to the self-reported response format. The fact that the study involved only patients with RRMS might be a limitation considering that patients with progressive MS have higher disability and larger impact on daily activities/work, compared with RRMS [52]. However, considering that patients with RRMS are the “active” subgroup of MS, this is possibly the subpopulation of greatest interest and with largest room for improvement in the clinical practice. The survey may possibly under-represent the RRMS population, due to age-related limitations (e.g., extremely severe cases of RRMS elderly patients are less likely to complete the survey) and limited access to the internet (e.g., very low-income individuals and elderly RRMS patients may not have computer access). A drawback with the matched sample is that the groups may differ on unmeasured variables that may have an impact on outcomes. The respondents’ population may not have been normally distributed which is evidenced by high standard deviation values. Job type or occupational characteristics were not considered in the analysis. The level of unemployment in both the RRMS and controls groups was higher than expected. The most current estimates from 2018 show the 55 years and over age group to have about 3% unemployment [53]. Work productivity may be influenced by the type of work. Physically and cognitively demanding jobs are associated with different rates of work impairment.

## Conclusion

In conclusion, among employed individuals, RRMS attributed to reductions in work productivity, including presenteeism and absenteeism compared with non-MS individuals indicating higher burden. Decrease in work productivity and increase in presenteeism and absenteeism was also associated with increased severity of work impairment. This study demonstrated that greater productivity loss is proportional to greater HCRU and lower HRQoL. The findings of the study suggest that reducing RRMS symptoms could potentially reduce associated burden and work force impact. This is especially important in the context of RRMS as individuals are often diagnosed in early to middle adulthood when they are part of the workforce. The impact of RRMS on work should be a consideration and point of discussion with newly diagnosed RRMS individuals who are considering recommendations for early treatment with MS DMTs in effort to slow disability accumulation.

## Supplementary information

**Additional file 1: Figure S1.** Respondent Flow Diagram. NHWS, National Health and Wellness Survey; MS, Multiple Sclerosis; RRMS, Relapsing-Remitting Multiple Sclerosis.

## Data Availability

Not applicable.

## Abbreviations

CCI: Charlson comorbidity index
CIS: Clinically isolated syndrome
ER: Emergency room(s)
GP: General physician
HCP: Healthcare providers
HCRU: Healthcare resource utilization
HRQoL: Health-related quality of life
MCS: Mental component summary
MID: Minimally important differences
MS: Multiple sclerosis
NHWS: National Health and Wellness Survey
PCP: Primary care physician
PCS: Physical component summary
RRMS: Relapsing-remitting multiple sclerosis
WPAI-GH: Work Productivity and Activity Impairment-General Health
