# Supplementary figures and images for: Burden of relapsing-remitting multiple sclerosis on workers in the US: a cross-sectional analysis of survey data
Source: BMC Neurol. 2019 Oct 28;19:258. doi: 10.1186/s12883-019-1495-z (PMC6816180; doi:10.1186/s12883-019-1495-z)

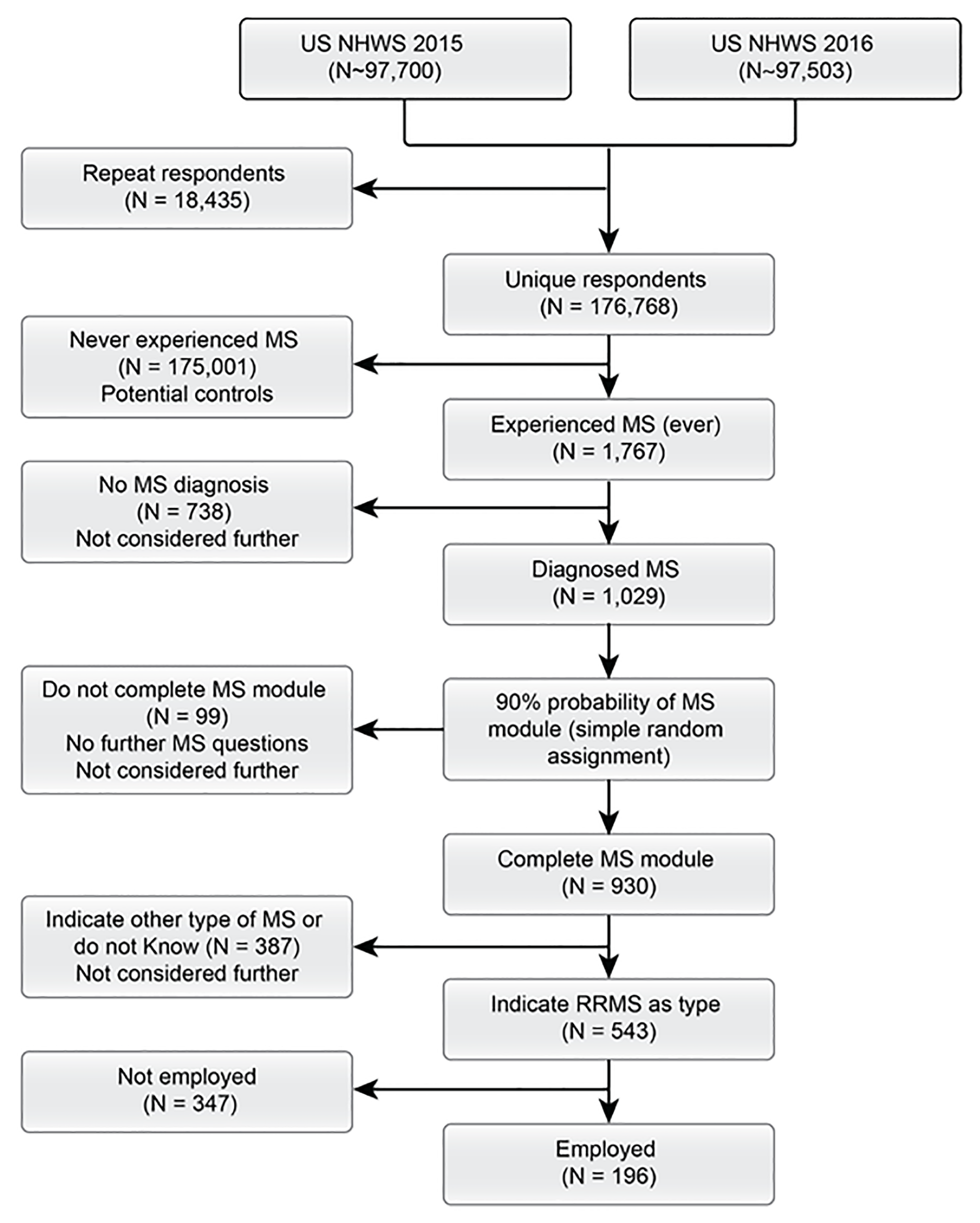

Supplement: Supplementary file 1 — Additional file 1: Figure S1. Respondent Flow Diagram. NHWS, National Health and Wellness Survey; MS, Multiple Sclerosis; RRMS, Relapsing-Remitting Multiple Sclerosis. [file 12883_2019_1495_MOESM1_ESM.tif]
